# Effects of electrochemical properties of ferrocenylpyrazolylnickel(ii) and palladium(ii) compounds on their catalytic activities in ethylene oligomerisation reactions†

**DOI:** 10.1039/c7ra13588b

**Published:** 2018-01-31

**Authors:** Collins Obuah, Michael K. Ainooson, James Darkwa

**Affiliations:** Department of Chemistry, University of Johannesburg Auckland Park Kingsway Campus, Auckland Park 2006 Johannesburg South Africa; Department of Chemistry, University of Ghana Legon Accra Ghana cobuah@ug.edu.gh

## Abstract

Palladium complexes of ferrocenylpyrazolylpyridine and ferrocenylpyrazolylamine were synthesised and screened as pre-catalysts (1–4) for olefin polymerisation. The pre-catalysts 1–4 on activation with EtAlCl_2_ in the presence of ethylene with chlorobenzene or hexane as solvent were highly active with 1 being the most active, with an activity of 360 kg mol Pd^−1^ h^−1^. The major product from the reaction was 1-butene and high carbon content oligomers. The molecular weight (*m*/*z*) of the high carbon content oligomers is as high as 623.0. When toluene is used as solvent, the products obtained were ethyltoluene and butyltoluene and 1-butene. The electronic properties of the ligands (L1–L7) and complexes (1–10) were determined by cyclic voltammetry (CV) and molecular modelling. The CV results show that the ferrocenyl is easily oxidized upon the introduction of pyrazolyl derivatives, the process is quasi-reversible. However, complexation of the ligands with palladium or nickel results in difficulty in oxidizing the ferrocenyl moiety. This is an indication of the electrophilic nature of both the palladium and nickel centres. The mechanism of the oxidation was observed to be diffusion-controlled and is independent of scan rate. Molecular modelling experiments show that nickel and palladium complexes have lower HOMO–LUMO gaps and high global descriptors, an indication of a highly electrophilic metal centre. A plot of the electrophilicity indices of the pre-catalysts against yield of the oligomers show a linear correlation, an indication that the electrophilicity of the metal centre plays an important role in the activity of these pre-catalysts.

## Abstract 2

Studying the correlation between the electrophilicity of the metal centre and the activity of the resulting catalyst using electrochemistry and computation modelling.

## Graphical abstract

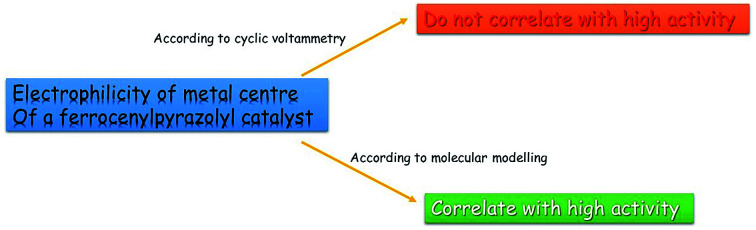

## 1. Introduction

Ethylene is the cheapest and most widely produced organic compound in the world,^1,2^ and is also an abundant feedstock for the manufacturing of value-added products including linear α-olefins (LAOs), a class of commodity chemicals that are used in industries such as synthetic polymers, detergents, plasticizers, and lubricants, and have a global demand of over four million tons per year.^3^ The synthesis of these commodity chemicals involves the use of suitable catalysts. The catalyst has to be fine-tuned for high activities. Fine-tuning of the catalyst involves the control of the electrons in the catalytic system through ligand design.

The synthesis of functionalised ferrocene is of great importance in the field of organometallic chemistry,^4^ this can be attributed to the unique properties of ferrocene and how functional groups attached changes the chemistry of ferrocene. Due to the robustness of ferrocene, many applications for its use exist, including its use as redox-active component in homo- and heterometallic transition metal complexes.^4^ Ferrocene and its derivatives have been used as molecular sensors,^5^ in energy transfer processes^6^ and as catalysts in various chemical reactions^7^ because of their reversible electrochemical nature. The redox reaction between the ferrocene and ferrocenium ion (Fe^II^/Fe^III^) is a fast and reversible one electron transfer process. This is one of the vital properties of ferrocene and its derivatives. Scientists have taken advantage of this property to focus on the electronic and communication properties in these compounds^8^ through electrochemical studies using techniques such as cyclic voltammetry (CV).^9,4*b*^ CV is a very useful electroanalytical technique for compounds containing transition metal centres that may take on several different oxidation states. The CV experiment using the ferrocene as proxy can provide important information about the electron density of transition metal centres in a compound as well as the compound's stability under the experimental conditions employed.

Computational modelling, on the other hand, is another technique that can be used to predict the electronic nature of compounds. Recent developments in the area of olefin oligomerisation and polymerisation catalysis using group 10 transition metal catalysts, mainly nickel(ii) and palladium(ii) catalysts, have shown that the electronic nature of the catalysts is crucial.^10^ To obtain the desirable catalysts requires not only synthetic skills but also an understanding of all kinds of factors influencing the fundamental steps of olefin oligomerisation and polymerisation reactions.^11^ There are numerous articles dealing with polymerisation catalysts of late-transition metals with N^O or N^N ligand systems. The more established N^N catalysts have been the subjects of numerous mechanistic studies based on experimental and theoretical techniques.^12^ The catalytic activity of the transition metal complex is relies more on the electronic configuration of the catalyst in the ground state, especially the charge density. Möhring *et al.*^13^ have shown that the electronic effect could contribute as much as 80% of the change in the polymerisation activities. This is by studying the influence of steric and electronic effect of ligands on the catalytic activity of (CpR)_2_ZrCl_2_/ethylaluminoxane. Guo *et al.* have also shown that metallocene catalyst's activities increase with the reducing charge density^14^ while the catalytic activities of α-diimine nickel(ii) complexes increase with increase charge density.^15^ Other report using Brookhart's type catalysts have demonstrated through theoretical mechanistic studies using quantum mechanics the importance of the conformation of the substituted on the catalysts and the electrophilicity of the central metal toward the activity of the catalysts.^16^ A part from theoretical and experimental evidence, other analytical methods to determine the electronic properties of catalysts have not been extensively investigated.

In view of the importance of electronic properties on catalysts on activity, this report deals with two simple analytical methods (electrochemical and computational modelling) to study the electronics of ferrocenylpyrazolyl nickel(ii) and palladium(ii) complexes. The ferrocene in the complexes is to service as a probe for the electrochemical studies using CV to indirectly determine the electrophilicity of the metal centre of the catalyst and relate it to the activity of the catalyst towards ethylene oligomerisation and polymerisation reactions.

## 2. Experimental section

### 2.1 Materials and instruments

Compounds were synthesised using standard Schlenk techniques under N_2_. Organic solvents such as *N*,*N*-dimethyl formamide (DMF) were dried over CaH_2_. Hexane over Na lamps while tetrahydrofuran (THF) was dried over Na with benzophenone as indicator. Compounds L1–L7 and 1–10 were synthesised according to literature procedure.^17^*Tetra-n*-butylammonium-tetrafluoroborate ([^*t*^BuN][BF_4_]) was purchased from Sigma Aldrich and used as received. All electrochemical experiments were performed using Autolab potentiostat PGSTAT 302 (Eco Chemie, Utrecht, The Netherlands) driven by the general purpose Electrochemical System data processing software (GPES, version 4.9). Infrared (IR) spectra of complexes were recorded on a Bruker Tensor 27 equipped with a diamond ATR. Elemental analyses were performed on a Vario elementar III microcube CHNS analyzer. The ESI-MS spectra were recorded on a Waters API Qualtro micro spectrophotometer. NMR spectra were recorded on a Bruker 400 MHz instrument (^1^H at 400 MHz and ^13^C{^1^H} at 100 MHz). The chemical shifts are reported in *δ* (ppm) and referenced to the residual proton and carbon signals 7.24 ppm and 77.0 ppm respectively of CDCl_3_ NMR solvent.

### 2.2 Catalysis

#### 2.2.1 General procedure for ethylene oligomerisation reactions

In a typical experiment, a 500 mL steel autoclave was heated under vacuum for 2 h at 160 °C and allowed to cool to the required temperature. The appropriate solution of the catalyst precursor for each reaction was first introduced into the reactor and the reaction mixture saturated with ethylene at 1 bar for 5 min and ethylene vented off. The co-catalyst, EtAlCl_2_ was added to the reaction mixture *via* a cannula. The reactor was then saturated with ethylene to the desired pressure and stirred at the required temperature and time. The ethylene pressure was maintained throughout the reaction. The reaction was stopped by turning off ethylene feed and connecting the reactor to two traps cooled with liquid nitrogen, followed by venting off the reactor. The reactor contents were isolated and quenched with 10% HCl in methanol, and the product sampled for gas-chromatography (GC) and gas-chromatography-mass spectrometry (GC-MS) analyses. The solvent from the remaining product was removed *in vacuo* and the mass of the product determined.

### 2.3 Electrochemical methods

The electrochemical cell consists of a three-electrode cell, which involved a glassy carbon (geometric area = 0.071 cm^2^) as a working electrode, Ag/AgCl (3 M KCl) reference electrode, and platinum wire as the counter electrode. The measurement was performed using a concentration of 1.0 mM samples, in the presence of 0.1 M *tetra*-butyl ammonium tetrafluoroborate ([^*t*^BuN][BF_4_]) (supporting electrolyte) in dry DMF under argon atmosphere at room temperature. The glassy carbon electrode was polished on a Buehler-felt pad using alumina (0.05 μm) and then washed with millipore water, sonicated for 5 min in millipore water and finally rinsed with pH 9.2 buffer solution. This was done to ensure that there are no contaminants on the electrode surface before the electrochemical measurements. Argon was bubbled through the solution before recording the cyclic voltammogram to ensure an oxygen free solution.

### 2.4 Molecular modelling method

Modelling programs Spartan 08 V1.2.0 and Spartan 10.1.10 (ref. 18) were used for all calculations at the DFT B3LYP level of theory. The B3LYP functional level of theory is a Hartree–Fock DFT hybrid. However, the LACVP* basis set is a relativistic effective core-potential or pseudo potential that describes the atoms H–Ar and transition metals with the 6-31G* basis. The LANL2DZ basis set was used to model heavier atoms which make use of all-electron valence double zeta basis set D95V, done by Dunning, for first row elements^19^ and the Los Alamos ECP plus double zeta basis set for the atoms Na–La, Hf–Bi^20^ done by Wadt and Hay. Spartan's graphical model builder and minimised which interact with the SYBYL force field^21^ were used to construct the starting geometries of the molecular systems. All geometries were fully optimised without any symmetry constraints. Equilibrium geometries were characterised by the absence of imaginary frequencies, which also gave the HOMO, LUMO and charge density values. The transition state structures were with only one imaginary frequency and located without constrains using fully optimised standard transition state optimisation procedure in Spartan.

## 3. Results and discussions

### 3.1 Catalysis

#### 3.1.1 Ethylene oligomerisation or polymerisation reactions using pre-catalysts 1–4

The ethylene catalysis of pre-catalysts 5–10 have been reported in literature by us.^17*b*,*c*^ We report here the extensive catalytic activity of pre-catalysts 1–4.

The ethylene oligomerisation or polymerisation reactions for pre-catalysts 1–4 were investigated. Pre-catalyst 1 was used as a basis to investigate the optimum co-catalyst ratio and pre-catalyst loading needed for the polymerization reaction in hexane. The co-catalyst used is EtAlCl_2_, whiles the Al : Pd ratio was varied from 100 : 1 to 200 : 1. This led to an increase in both the activity of the catalyst and molecular weights of the polyethylene produced. However, increasing in the ratio above 200 : 1 was followed by a drastic decrease in both activity and molecular weight. The decrease in molecular weight on increasing the Al : Pd ratio is an indication of high chain transfer from the palladium species to the aluminium co-catalyst and fast chain termination, this in line with what is reported in the literature.^22^

The effect of catalyst loading of pre-catalyst 1 was also investigated. It was observed that increasing the pre-catalyst loading from 4 μmol to 5 μmol resulted in an increase in activity from 200 kg mol Pd^−1^ h^−1^ to 262 kg mol Pd^−1^ h^−1^. A further increase in pre-catalyst amount to 10 μmol, caused the activity to drop to as low as 131 kg mol Pd^−1^ h^−1^. However, the molecular weight of the polymer remained unchanged. A similar observation has been reported by Junges *et al.*^23^ for a tridentate pyrazolyl Cr(iii) pre-catalyst. In this system, a pre-catalyst loading of 10 μmol gave a TOF of 66 200 h^−1^ but at 30 μmol loading reduces the TOF to 29 000 h^−1^. Junges *et al.* attributed this to the ease at which the pre-catalyst solubilised in the reaction solvent at low pre-catalyst loading and catalyst aggregation at high pre-catalyst loading.

**Scheme 1:**
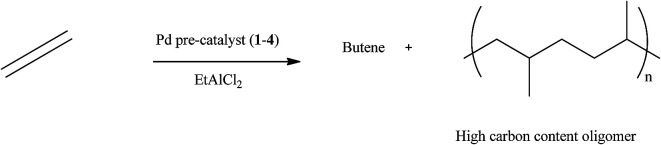
Oligomerisation of ethylene catalysed by pre-catalyst 1–4 with EtAlCl_2_.

The optimum Al : Pd ratio was 200 : 1 and pre-catalyst loading of 5 μmol was established. Further catalytic investigations were performed in ethylene oligomerisation and polymerisation reactions using EtAlCl_2_ as co-catalyst (Scheme 1). At these optimum conditions other reaction parameters such as ethylene pressure, temperature, solvent and time were investigated.

The oligomerisation reaction was not exothermic, as there was no temperature increase during the reaction. All the pre-catalysts show moderate activities as ethylene oligomerisation catalysts to produce mostly butene and higher chain oligomers. A typical GC (Fig. S1†) shows the presence of butene, which is 1-butene but did not show the presence of higher oligomers. However, the product obtained after removing the solvent was a viscous liquid, which was initially suspected to be a low molecular weight polymer. GPC analyses were performed on these liquid products, their molecular weights were found to be lower than 1000 Da. Using Atmospheric pressure chemical ionization (APCI) a soft ionization technique, the molecular weights observed were between 267.0 and 623.0, an indication that the viscous liquid products are oligomers of ethylene. This technique gave an envelope shape spectra, indicating a single active catalytic species present upon activation with EtAlCl_2_ co-catalyst. A typical spectrum of the APCI is shown in Fig. S2.† An expanded form of the spectrum which shows isotopic distributions is presented in Fig. S3.† The difference between the isotopic patterns is 14.0157 which correspond to a methylene group, indicating fragmentation of the oligomer chain.

^1^H NMR spectrum (Fig. S4†) shows an olefinic group that support the low molecular weight of the ethylene oligomer produced. The olefinic group, appearing at 5.21 ppm is a vinylene group (*E* and *Z* R^1^CH=CHR^2^). The peaks appearing between 0.80 ppm and 2.10 ppm are different protons from CH_2_ and CH_3_ groups indicating that the products obtained in the catalytic reactions are ethylene oligomers.

#### 3.1.2 Effect of catalyst structure on the catalytic activity

Among the six pre-catalysts investigated pre-catalyst 1 was found to be the most active (Table 1: entries 1–6). It appears the nature of the ligands play a significant role in the catalyst activity and the molecular weight of the ethylene oligomers. Pre-catalysts 1 and 2 that have pyrazolyl-pyridyl donor groups were more active and produced polymers with the highest molecular weights compared to 3 and 4 that have pyrazolyl-amine donor groups. For instance, under the same reaction conditions, the best activity for the pre-catalyst containing pyrazolyl-pyridyl ligands (1 and 2) was 262 kg mol Pd^−1^ h^−1^ with a corresponding molecular weight of 507.0 for 1. The activity for pre-catalyst with pyrazolyl-amine donors (3 and 4) was 210 kg mol Pd^−1^ h^−1^ with 401.0 molecular weight for 3. The high activities of pre-catalysts 1 and 2 compared to 3 and 4 could be due to the stable pre-catalysts formed by the strong donor ability of the pyridyl unit. Also pre-catalysts 1 and 3 that show high activities compared to 2 and 4. These have no substituent in position 3 of the pyrazolyl unit. This is an indication that steric factors play a crucial role in the activity of these pre-catalysts. Due to the high catalytic activity of pre-catalyst 1, it was used in further studies on various catalytic reaction parameters.

**Table 1:** Ethylene oligomerisation reaction using pre-catalysts 1–4 and EtAlCl_2_ as co-catalyst^a^

Entry	Catalyst	Pressure (bar)	Yield^d^ (g)	Activity (kg mol Pd^−1^ h^−1^)	Temperature (°C)	Time (min)	Oligomer weight^e^ (*m*/*z*)
3	1	10	1.31	262	25	60	507.0
4	2	10	1.05	210	25	60	425.0
5	3	10	0.98	196	25	60	401.0
6	4	10	0.65	130	25	60	345.0
7	1	5	0.66	132	25	60	301.0
8	1	20	1.41	282	25	60	511.0
9	1	30	1.56	312	25	60	520.0
10	1	10	0.90	180	40	60	302.0
11	1	10	0.61	122	50	60	243.0
12	1	10	0.56	112	60	60	220.0
13	1^b^	10	1.65	330	25	60	—
14	1^c^	10	1.81	362	25	60	623.0
15	1	10	1.46	146	25	120	512.0
16	1	10	1.58	105	25	180	519.0

a Conditions: 6.0 mL of hexane.

b Toluene used as reaction solvent.

c Chlorobenzene used as reaction solvent; catalyst loading = 5 μmol; Al : Pd = 200 : 1.

d Determined by mass difference of 70.0 mL hexane (45.8 g), toluene (60.9 g) or chlorobenzene (77.7 g) and mass of final solution.

e Determined by APCI.

Kinetically, the rate of oligomerisation or polymerisation increases with increase in ethylene pressure.^24^ The activity of pre-catalyst 1 and molecular weight of the ethylene oligomers produced showed marginal increment with increase in pressure. As an illustration, increasing the pressure from 10 bar to 30 bar saw only 0.10 g and 0.25 g increase from 1.31 g of products formed and also slight increase in activity (Table 1: entries 3, 8 and 9). A similar trend was observed for the molecular weights recorded. Where pressure is increased from 10 bar to 30 bar resulted in molecular weight of 507.0, 511.0 and 520.0 respectively (Table 1: entries 3, 8 and 9). The marginal increase in both mass and molecular weight as pressure was increased could be due to catalyst saturation.

Temperature variation affects the activity of the catalysts and molecular weight of the polymer obtained. For example an increase in temperature from 25 °C to 60 °C resulted in a decrease in activity from 262 kg mol Pd^−1^ h^−1^ to 112 kg mol Pd^−1^ h^−1^ (Table 1: entries 3, 10–12). This can be attributed to decomposition or deactivation of the active species as well as lower ethylene solubility in the reaction solution at high temperatures.^25^ The decrease in molecular weight could be due to faster chain termination than chain propagation at elevated temperature.^26^

The reaction solvent was also varied by using hexane, toluene and chlorobenzene respectively. An increase in activity is observed which can be attributed to solubility of the active species (Table 1: entries 3, 13 and 14). The results show that the active species is more soluble in a polar solvent, hence the observed increase in activity in the order hexane < toluene < chlorobenzene. Butene and liquid polyethylene were obtained when hexane and chlorobenzene were used as reaction solvent. The higher molecular weight of 623.0 was obtained for the oligomer using chlorobenzene, compared to 507.0 obtained for the reaction in hexane under the similar reaction conditions. In toluene, the products obtained were butene, ethyltoluene, mono-butyltoluenes and di-butyltoluenes (Fig. S5†). The alkyltoluenes are produced *via* Friedel–Crafts alkylation reaction.^27^ The oligomerisation reactions performed in toluene resulted in alkylation of the solvent with the oligomer produced and the ethylene. There was no clear evidence of triple alkylation of toluene from the GC data (Fig. S5†); suggesting that alkylation is more facile for butyltoluenes compared to ethyltoluenes.

Time run experiments were performed to establish the life time of the active species. Increasing the time 60 min to 180 min saw only a marginal increase in catalytic activity and molecular weight (Table 1: entries 15 and 16). After 60 min catalytic activity was low towards polymerization. The low activity recorded with longer reaction time indicates catalyst deactivate with time.

The following sections deal with extensive report on the determination of the electronic properties of the ligands and the complexes using CV and computational modelling.

### 3.2 Electrochemical studies

#### 3.2.1 Electrochemical properties of compounds L1–L7

At a given temperature, the half-wave potential (*E*_1/2_) values change considerably for different solvents. The half-wave potential shifts toward more positive potentials in the order: DMSO < DMA < NMF < DMF < ACN < PEN < ACE < DCM. This order indicates that the oxidation of ferrocene to the ferrocenium is more difficult with decreasing polarity.^28^ The ferrocenium is more sensitive to interactions with the solvent molecules than ferrocene. Therefore, the strength of the interactions of the ferrocenium (which acts as a Lewis electron-pair acceptor) with solvent molecules (Lewis electron-pair donor) is expected to affect the oxidation of ferrocene and thus the half-wave potential.^29^ Based on the above hypothesis, *N*,*N*-dimethyl formamide (DMF) which is midway through the series was chosen as solvent for the cyclic voltammetry experiment.

**Fig. 1:**
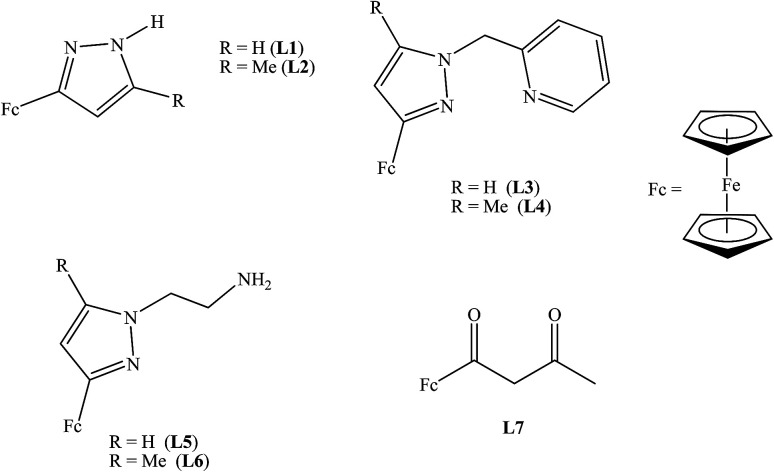
Ferrocenylpyrazolyl compounds L1–L7.

The electrochemical properties of ligands L1–L7 and palladium(ii) and nickel(ii) complexes 1–10 were investigated by cyclic voltammetry in DMF with 0.1 M [^*t*^BuN][BF_4_] as supporting electrolyte. Table 2 summarises the redox potential data for compounds L1–L7 investigated (Fig. 1).

**Table 2:** Cyclic voltammetry data for compounds L1–L7^a^

Entry	Compound	*E*_1/2_ (mV)	Δ*E* (mV)	*I*_pc_/*I*_pa_
1	Fc	483.0	84.0	0.85
2	L1	335.0	97.0	0.72
3	L2	362.0	101.0	0.75
4	L3	325.0	83.0	0.63
5	L4	329.0	97.0	0.74
6	L5	357.0	86.0	0.72
7	L6	332.0	87.0	0.68
8	L7	633.0	155.0	0.12

a Conditions: solvent = *N*,*N*-dimethyl formamide (DMF); supporting electrolyte = *tetra-n*-butylammonium-tetrafluoroborate [^*t*^BuN][BF_4_]; compound concentration = 1 mM; supporting electrolyte concentration = 0.1 M. Reference electrode = Ag/AgCl; counter electrode = platinum wire; working electrode = glassy carbon; Fc = ferrocene, scan rate of 100 mV s^−1^. Fc = ferrocene.

The cyclic voltammograms of compounds L1 and L2 showed a quasi-reversible peak corresponding to one electron transfer. The typical voltammogram for L2 is shown in Fig. 3. Fe^II^–Fe^III^ redox couples of the ferrocenyl unit of L1 and L2 showed *E*_1/2_ at 335 mV (Δ*E* = 97 mV, and *I*_pc_/*I*_pa_ = 0.72) and 362 mV (Δ*E* = 101 mV and *I*_pc_/*I*_pa_ = 0.75) respectively (Table 2: entries 2 and 3). The observed redox potentials are in agreement with the electron donor abilities of the pyrazolyl unit, which donates more electron density to the ferrocene moiety; and hence favouring the ease of oxidation of the ferrocenyl unit.^30^ Modification of L1 and L2 by introducing more electron donating groups such as methylene pyridine (L3 and L4) and ethylamine (L5 and L6) show significant improvement on the oxidation and still show quasi reversibility of the ferrocenyl unit (Table 2: entries 2, 3 and 4–7). However, L3 and L4 showed the oxidation of ferrocenyl unit is easier compared L5 and L6. This could be due to the aromatic nature of the pyridine ring which helps to form a partial conjugation system with the pyrazolyl unit. Compared to compounds containing ethylamine group, despite its electron donating ability, these ligands do not form a conjugated system; hence this has less oxidative influence compared to the methylene pyridine. The influence of substituent (either hydrogen or methyl) on the pyrazolyl moiety also appears to play a role in the oxidation of the ferrocenyl unit. In principle, the electron donating methyl substituents should enhance the ease of oxidation compare to hydrogen. Comparing the compounds which differ by either methyl or hydrogen on the pyrazolyl unit, only L6 undergoes easy oxidation compared to L5. Compounds L1, L2, L3 and L4 however, showed otherwise. As expected, compound L7 which has an electron withdrawing group shows one electron quasi-reversible oxidation peak (Fig. S6†) of the ferrocenyl unit with half wave potential of 633.0 mV and *I*_pc_/*I*_pa_ value of 0.12 (Table 2: entry 8).

**Fig. 2:**
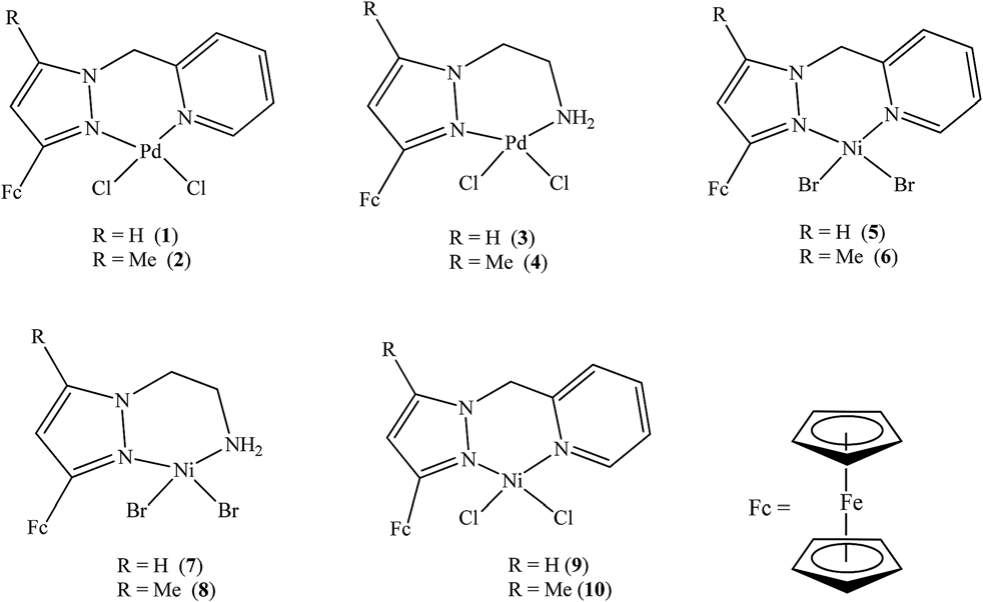
Ferrocenylpyrazolyl palladium and nickel complexes 1–10.

**Fig. 3:**
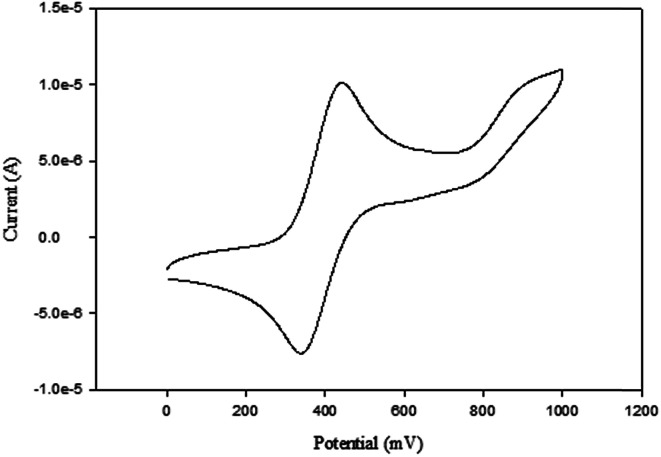
Cyclic voltammogram of compound L2 in DMF, [^*t*^BuN][BF_4_] as supporting electrolyte, Ag/AgCl as reference electrode, platinum wire as counter electrode, glassy carbon as working electrode, scan rate of 100 mV s^−1^, temperature 25 °C.

A plot of current (A) against scan rate (mV s^−1^) show a linear relationship for all the compounds discussed above. The linear relationship suggests that the mechanisms of oxidation of the ferrocenyl unit in L1–L7 are diffusion controlled. A typical plot for L2 is shown in Fig. S7.† The diffusion controlled mechanism indicates that there is a spontaneous transfer of the electroactive species from regions of higher concentrations to regions of lower concentrations.

The compounds also show that the half wave potentials are independent of scan rates. An example is given in Fig. S8,† which shows half wave potentials are independent of scan rates for compound L2. This also demonstrates that the diffusion controlled process occurs quickly, and the rate of reaction is controlled by rate of transport of the reactants through the medium.

#### 3.2.2 Electrochemical properties of complexes 1–10

**Fig. 4:**
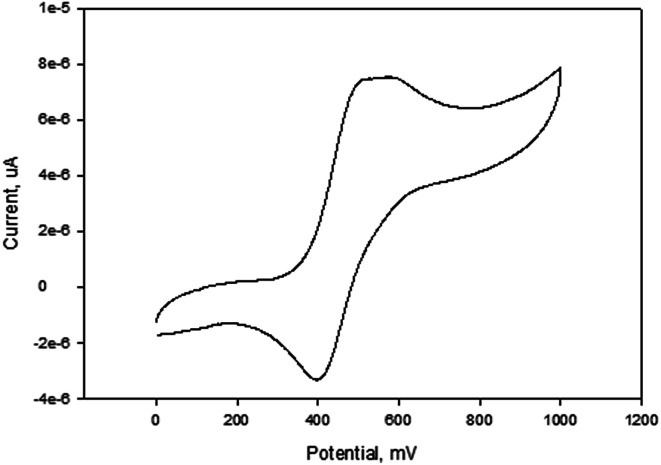
Cyclic voltammogram of complex 1 in DMF, [^*t*^BuN][BF_4_] as supporting electrolyte, Ag/AgCl as reference electrode, platinum wire as counter electrode, glassy carbon as working electrode, scan rate of 100 mV s^−1^, temperature = 25 °C.

Palladium complexes 1–4 showed redox potential for the ferrocenyl unit with higher *E*_1/2_ values between 410 and 456 mV (Table 3) compared to that of the corresponding ligands which are between 325 and 357 mV. Also, the observed *I*_pc_/*I*_pa_ values were between 0.40 and 0.49, which is an indication of also quasi-reversible behaviour. This could be due to the dative covalent bonds which are formed between the palladium and the nitrogen donor atoms of the ligand. Therefore, electrons are donated from the ligand to the palladium metal centre, resulting in low electron density on the ferrocenyl moiety. Hence, the palladium complexes showed higher half wave potential for Fe^II^/Fe^III^ redox couple than their corresponding ligands. A typical voltammogram for complex 1 is shown in Fig. 4. There is no evidence from the voltammogram of delocalisation of electrons between the two metal centres. In this voltammogram, the second wave potential is difficult to determine. This could suggest aggregation of the complexes or lack of interaction between the two metal centres.

**Table 3:** Cyclic voltammetry data for complexes 1–10^a^

Entry	Compound	*E*_1/2_ (mV)	Δ*E* (mV)	*I*_pc_/*I*_pa_	*K*_c_	Yield
1	1	456.0	118.0	0.44	—	1.31
2	2	412.0	104.0	0.46	—	1.05
3	3	423.0	120	0.40	—	0.98
4	4	410.0	115	0.48	—	0.65
5	5	425.0	92.0	0.46	589	17.80^b^
6	6	393.0	95.0	0.49	613	10.20^b^
7	7	436.0	83.0	0.49	130	6.70^b^
8	8	433.0	77.0	0.72	170	5.80^b^
9	9	417.0	81.0	0.50	520	8.20^b^
10	10	424.0	95.0	0.35	592	7.10^b^

a Conditions: solvent = *N*,*N*-dimethyl formamide (DMF); supporting electrolyte = *tetra-n*-butylammonium-tetrafluoroborate [^*t*^BuN][BF_4_]; analyte concentration = 1 mM; supporting electrolyte concentration = 0.1 M. Reference electrode = Ag/AgCl; counter electrode = platinum wire; working electrode = glassy carbon, scan rate of 100 mV s^−1^.

b Published results from ref. 17*b*.

**Fig. 5:**
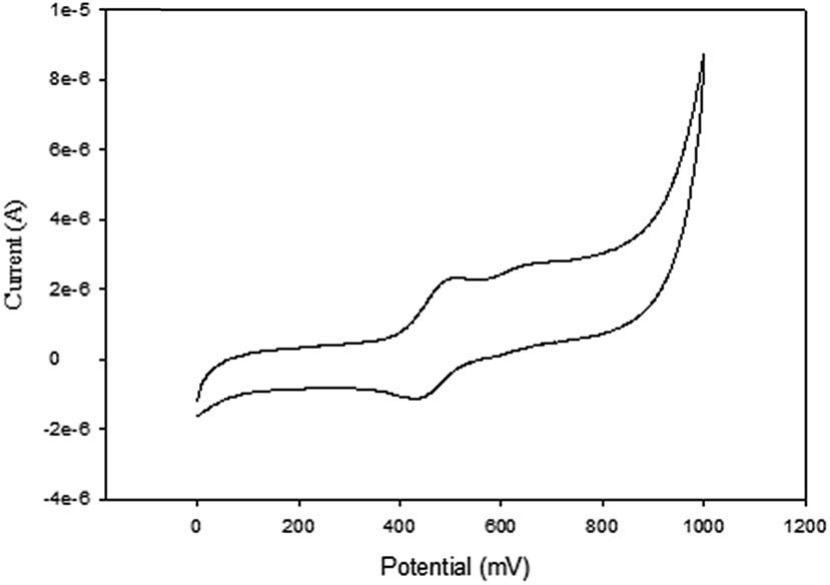
Cyclic voltammogram of complex 6 in DMF, [^*t*^BuN][BF_4_] as supporting electrolyte, Ag/AgCl as reference electrode, platinum wire as counter electrode, glassy carbon as working electrode, scan rate of 100 mV s^−1^, temperature 25 °C.

NiBr_2_ or NiCl_2_ complexes of L1–L6 (5–10 (Fig. 2)) also showed redox oxidation potential of the ferrocenyl unit in the various ligands. The half wave potentials for these complexes were between 389 and 436 mV and are higher than corresponding free ligands (Table 3). However, these half wave potentials are still lower compared to ferrocene (483 mV). The *I*_pc_/*I*_pa_ values also show that the oxidation of these complexes are quasi-reversible. The voltammograms of the complexes show delocalisation of electron between the metal centres. A typical voltammogram showing delocalisation of electron is Fig. 5. The extent of electron exchange between the iron and nickel centres could be expressed by the comproportionation constant (*K*_c_) using the Nernst equation.^31^*K*_c_ = exp(Δ*E*)*F*/*RT*(Δ*E* = *E*_2_ − *E*_1_, *F* = Faraday constant, *R* = gas constant, *T* = temperature).

To compare the degree of interactions between the metal centres using their equilibrium constants, the Robin and Day classification scheme was used.^32^ The Robin and Day classification scheme classifies molecules into three different classes. A class I molecule has the least amount of electronic interaction with a value *K*_c_ < 10^2^. Class II molecules have moderate electronic interactions, ranging between 10^2^ and 10^6^. Class III molecules on the other hand have *K*_c_ > 10^6^. Class III molecules have the best electron interactions. The degree of electronic interaction is directly related to the rate of the electron transfer *i.e.* large electronic interaction prefers fast electron transfer rates, while small electronic interaction depicts slow electron transfer rates.

The comproportionation constants *K*_c_ for complexes 5–10 (Table 3) suggests moderate delocalisation of electrons between the two metal centres. Complexes 7 and 8 recorded the lowest comproportionation constants, compared to 5, 6, 9 and 10, which could be due to the non-aromatic nature of the ligands, hence reducing the extent of electron transfer through conjugation. Complexes 5–10 therefore can be classified under the class II, according to the Robin and Day classification scheme.

The higher the half wave potential the more electrophilic the metal centres it is associated with. This indicates that the higher the half wave potential for Fe^II^/Fe^III^ redox couple, the difficult will it be to oxidise the ferrocenyl unit due to the withdrawal of electron density by nickel(ii) or palladium(ii) unit. In this way, we used ferrocene as a sensor to probe the electrophilic nature of both the nickel and palladium complexes. We have also correlated the electrophilicity of the nickel or palladium centre with their catalytic activities.

With the same ligand system, there is a direct correlation between the electrophilicity of the metal centre and yield. For example Table 3, entries 1–8, higher yields are associated with lower *E*_1/2_ values. In addition, Table 3, entries 5 and 9, with different halide substituents but the same metal and ligand environment, a similar relationship is observed. The nickel complexes generally have higher yields compared to palladium complexes and it is associated with lower *E*_1/2_ values. For example, in Table 3, entries 1 and 9, the nickel complex shows a higher yield compared to the palladium analogue. However, a similar observation was not made for entries 2 and 10. Other factors in addition to the electrophilicity of the metal centre are responsible for the high activities of the complexes. Other factors, such as solubility and stability of the active species can also play roles in the activities of these catalysts.

To further investigate the effect of the electrophilicity of the metal centre on activity and yield, molecular modeling was carried out.

### 3.3 Molecular modeling of ligands and complexes

#### 3.3.1 Electronic properties of ligands L1–L6

To investigate the electronic properties of these ligands; *ε*_HOMO_, *ε*_LUMO_, and global descriptors such as chemical potential (*μ*), chemical hardness (*η*) and electrophilicity (*ω*) were calculated according to the method used by Thanikaivelan *et al.*^33^ These descriptors are important electronic features used to describe stability, reactivity and other related properties of molecules.^34^ These descriptors were determined from HOMO and LUMO energies using the equations:
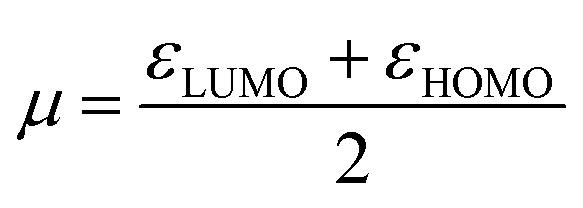

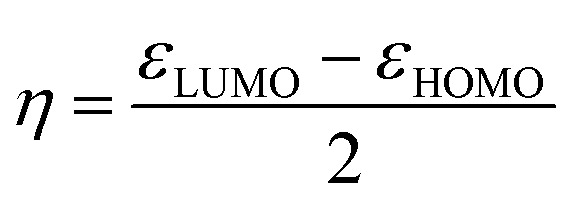

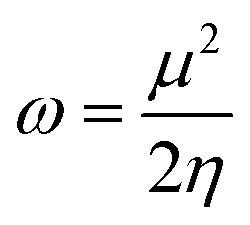


Generally, the HOMO of a ligand provides information about electron donating ability while the LUMO gives the electron accepting capability. Therefore, to understand the nature of the donor–acceptor behaviour the shape of the HOMO and LUMO can be used.

Fig. S9† shows that the HOMO of both L1 and L4 are localized on both the pyrazolyl nitrogen and the iron of the ferrocenyl unit. The localization of the HOMO on the pyrazolyl nitrogen atom is in agreement with the formation of σ-bond through that nitrogen atom to a metal. The HOMO of the iron metal is an indication of a possible transfer of the electron density to the pyrazolyl unit during the complex formation. The LUMO also shows localization on the pyridyl unit, which is an indication of potential π-acceptor behaviours of this arm of the ligand. However, L1 shows localization in the whole ligand system also suggesting that this ligand has π-acceptor potential. The strength of π-acceptor potential is determined by the more negative value of *ε*_LUMO_. Table 4 shows the values for *ε*_LUMO_ for ligands L1–L6. The values show that the ligands containing pyridyl unit have more electron accepting capabilities compared to the rest (Table 4: entries 3 and 4). This is followed by the aliphatic amines (Table 4: entries 5 and 6) then the pyrazolyl ligands (Table 4: entries 1 and 2).

**Table 4:** Data of global descriptors; chemical potential (*μ*), chemical hardness (*η*) and electrophilicity (*ω*) using B3LYP level of theory and LACVP* basis set for L1–L6 and 1–8

Entry	Compound	*ε*_HOMO_ (eV)	*ε*_LUMO_ (eV)	*μ* (eV)	*η* (eV)	*ω* (eV)	Yield (g)
1	L1	−5.15	−0.17	−2.66	2.49	1.42	
2	L2	−4.97	0.03	−2.47	2.50	1.22	
3	L3	−5.17	−0.71	−2.94	2.23	1.94	
4	L4	−5.12	−0.77	−2.95	2.18	1.99	
5	L5	−5.14	−0.65	−2.92	2.23	1.91	
6	L6	−5.09	−0.69	−2.89	2.20	1.89	
7	1	−5.33	−2.24	−3.79	1.55	4.63	1.31
8	2	−5.45	−2.14	−3.80	1.66	4.34	1.05
9	3	−5.28	−2.14	−3.71	1.57	4.38	0.98
10	4	−5.87	−2.12	−3.99	1.88	4.23	0.65
11	5	−5.25	−3.47	−4.36	0.89	10.68	17.80^a^
12	6	−5.23	−3.39	−4.31	0.92	10.10	10.20^a^
13	7	−5.30	−3.23	−4.27	1.04	8.77	6.70^a^
14	8	−5.41	−3.21	−4.31	1.10	8.44	5.80^a^

a Published result in ref. 17*b*.

To confirm further the π-accepting nature of these ligands, the electrophilicity index of each ligand was determined (Table 4). The higher the electrophilicity index the more electrophilic the compound. The data shows that the pyridyl ligands are more electrophilic than the ligands containing aliphatic amines, which are in turn more electrophilic than to the pyrazolyl ligands. Furthermore the ligands with hydrogen at the 3 position of the pyrazolyl unit are more electrophilic compared to the methyl substituted ligands.

##### Determination of the electronic properties of the complexes 1–8

The activity of metal pre-catalyst for olefin oligomerisation or polymerisation reactions, depend strongly on the nature of the ligands surrounding the metal. The nature of ligands in fact influences the electrophilicity of the metal centre. A highly electrophilic metal pre-catalyst, is expected to be highly active because the olefin is electron rich and therefore can readily coordinate to a more electrophilic metal centre.

HOMO–LUMO gap, global descriptors (chemical hardness (*η*) and electrophilicity (*ω*)) and charge density are the parameters considered in predicting the electrophilicity of the metal centres of these complexes.

#### 3.3.2 HOMO–LUMO gap

The HOMO–LUMO gap of a metal complex shows the direction of flow of electrons. Since the HOMOs are predominantly occupied by d-orbital electrons, smaller HOMO–LUMO gap will facilitate π-back donation of electrons from the metal to the ligand system and hence the metal centre will be electrophilic. The calculations performed on complexes 1–8 (Table 4; entries 7–14) show that the gaps for the palladium complexes are larger compared to the nickel complexes. This suggests that the nickel centres are more electrophilic compared to the palladium. Among the palladium complexes 1 (Table 4: entry 7) has the least gap while for the nickel complex 5 recorded the smallest gap (Table 4: entry 11). The results correspond to the experimental results where 1 and 5 were the most active among their respective complexes. Typical diagrams of the HOMO–LUMO gaps of complexes 2 and 6 are shown in Fig. S10 and S11.† It is therefore expected that the nickel complexes would be more active compared to the palladium complexes for olefin oligomerisation or polymerisation reactions. This is confirmed by experimental observations. The palladium complex 1 when activated with EtAlCl_2_ in the presence of ethylene shows an activity of 362 kg mol Pd^−1^ h^−1^ whereas the nickel complex 5 shows activity of 1776 kg mol Ni^−1^ h^−1^ under similar conditions.^17*b*^

### 3.4 Determination of the electronic properties of 1–8 using global descriptors

The global descriptors chemical hardness (*η*) and electrophilicity (*ω*) were used to predict the electronic properties of 1–8.

#### 3.4.1 Chemical hardness (*η*)

**Fig. 6:**
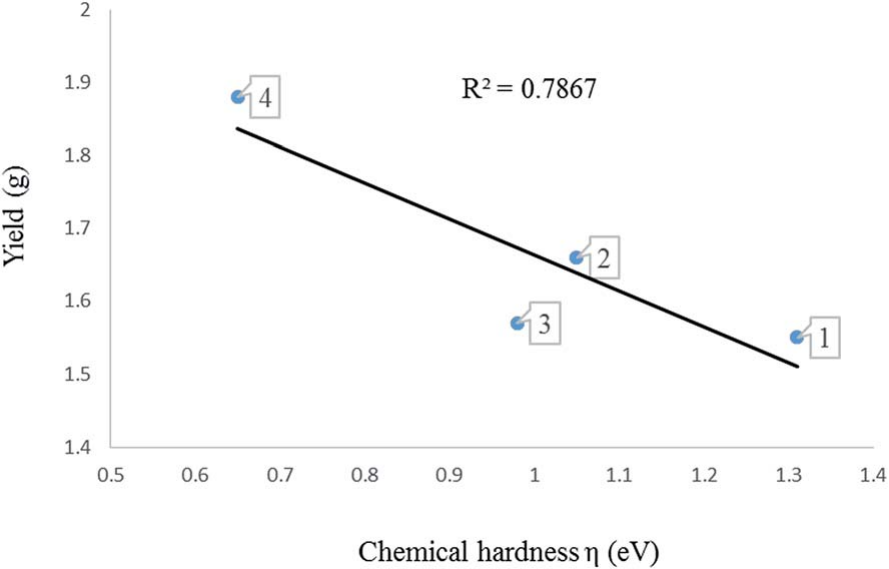
A graph of chemical hardness against yield of oligomers produced using complexes 1–4.

**Fig. 7:**
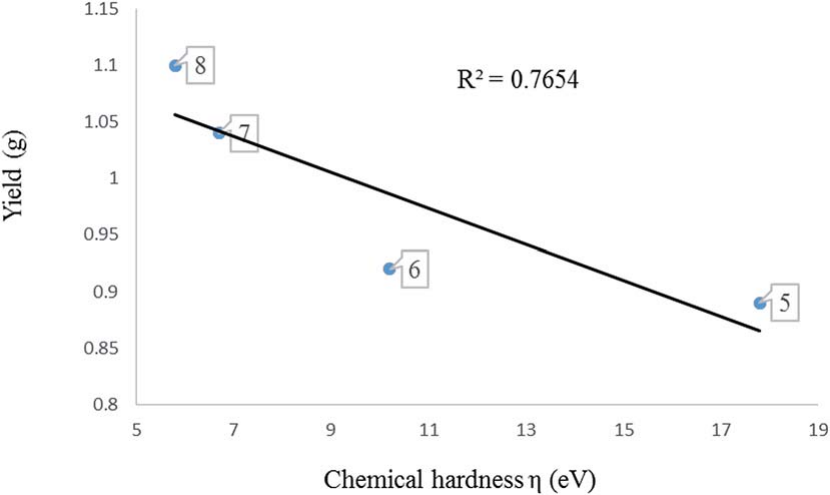
A graph of chemical hardness against yield of oligomers produced using complexes 5–8.

The chemical hardness (*η*) of a complex is an electronic quantity which is used to characterise the relative stability and the resistance to changes in the number of electrons.^35^ The higher the value for the chemical hardness the more stable and less reactive. The data in Table 4 entries 11–14 show that the nickel complexes have chemical hardness values between 0.81 and 1.65 eV which make them more reactive compare to the palladium complexes with values between 1.55 and 1.95 eV (Table 4; entries 7–10). In order to find a correlation between the calculated chemical hardness of the complexes and observed catalytic yield, a graph of yield against calculated chemical hardness was plotted (Fig. 6 and 7). The graph for the palladium complexes (Fig. 6) shows a near linear fit with *R*^2^ value of 0.7867, indicating a correlation between these two parameters. The best yield was obtained when the chemical hardness was minimum (1.55 eV) and the least yield was found at 1.88 eV. Similar observation was made for the nickel complexes (Fig. 7) where the *R*^2^ value is 0.7654 was recorded.

#### 3.4.2 Electrophilicity index (*ω*)

A quantitative classification of the global electrophilic nature of the complex can be determined by the electrophilicity index. The higher the value of *ω*, the more electrophilic the metal centre in the complex. Table 4 show that the nickel complexes are more electrophilic compared to the palladium complexes. To relate the catalytic yields of the complexes to electrophilicity index, a graph of electrophilicity index against yield were plotted as shown Fig. S12 and S13† for palladium and nickel complexes respectively.

#### 3.4.3 Charge density

The charge density of an atom or element shows the density of electrons located around the atom. The more positive the charge density the more electrophilic the atom or element is. The molecular modelling of the charge density for complexes 1–8 are presented in Table S2† respectively and the typical images of the charge densities for 1 and 6 drawn with isovalue of 1.0 are shown in Fig. S14 and S15.† The electron density values for both the palladium and nickel complexes are in agreement with the prediction made with the HOMO–LUMO gap. The palladium centres are less positive compared to the nickel centres.

## 4. Conclusion

The palladium complexes were used as pre-catalysts for the oligomerisation of ethylene upon activation with EtAlCl_2_ to 1-butene and higher carbon content oligomers, typically with 20 to 52 carbon atoms in the oligomer. The palladium pre-catalysts showed high activities in chlorobenzene for ethylene reactions than in hexane. In toluene, in addition to butene, ethyltoluenes and butyltoluenes were produced. The cyclic voltammetry studies show that ligands L1–L6 which have electron donating groups aid the ease of oxidation of the ferrocenyl moiety, while for L7 which has electron withdrawing group, oxidation of the ferrocenyl unit is difficult.

Cyclic voltammetry results for the nickel and palladium complexes show it is more difficult to oxidise the ferrocenyl unit in these ligands, indicating the electrophilic nature of both metals. A direct correlation between the electrophilicity of the metal is associated with a lower *E*_1/2_ and yield within a similar ligand system.

Molecular modelling of the palladium and nickel complexes show singlet state species and these complexes are stable compared to their corresponding ligands, which is expected. Calculations of the electronic properties of the complexes show that electrophilicity of the metal centre correlate with the activity of the pre-catalysts, an indication of the role electrophilicity plays in the activity of the pre-catalysts. Molecular modelling show that the higher the electrophilicity of the metal centre the high the activity of the catalyst.

This study shows that CV and molecular modelling studies could be used as simple tool to predict activity of a catalyst.

## Conflicts of interest

There are no conflicts to declare.

## Supplementary Material

RA-008-C7RA13588B-s001
