# Supplementary material for: Effects of electrochemical properties of ferrocenylpyrazolylnickel(ii) and palladium(ii) compounds on their catalytic activities in ethylene oligomerisation reactions
Source: RSC Adv. 2018 Jan 31;8(10):5362–71. doi: 10.1039/c7ra13588b (PMC9078115; doi:10.1039/c7ra13588b)
Supplement: RA-008-C7RA13588B-s001 [file RA-008-C7RA13588B-s001.pdf]

Electronic Supplementary Material (ESI) for RSC Advances.
This journal is © The Royal Society of Chemistry 2018

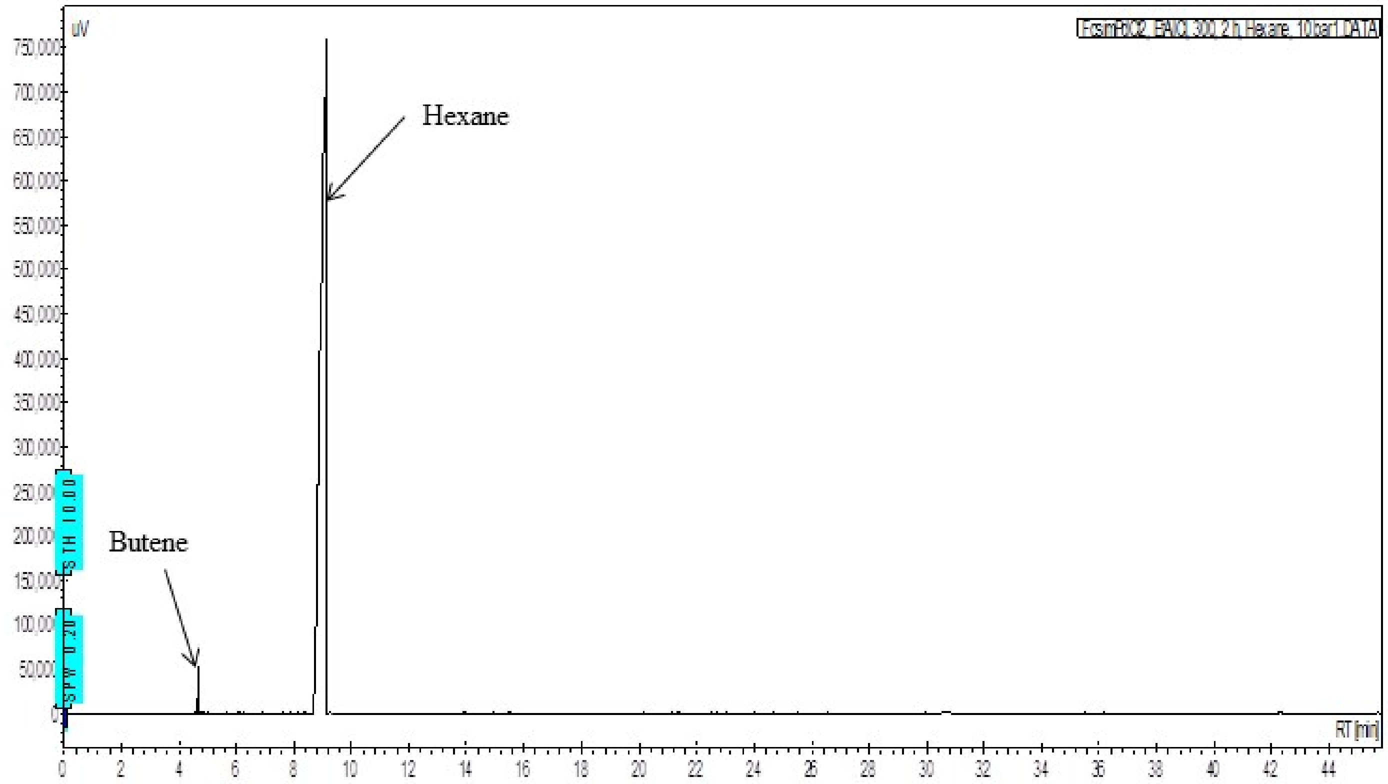

Figure S1: A typical GC showing the formation of 1-butene in ethylene oligomerisation reaction catalysed by pre-catalyst **1-4**

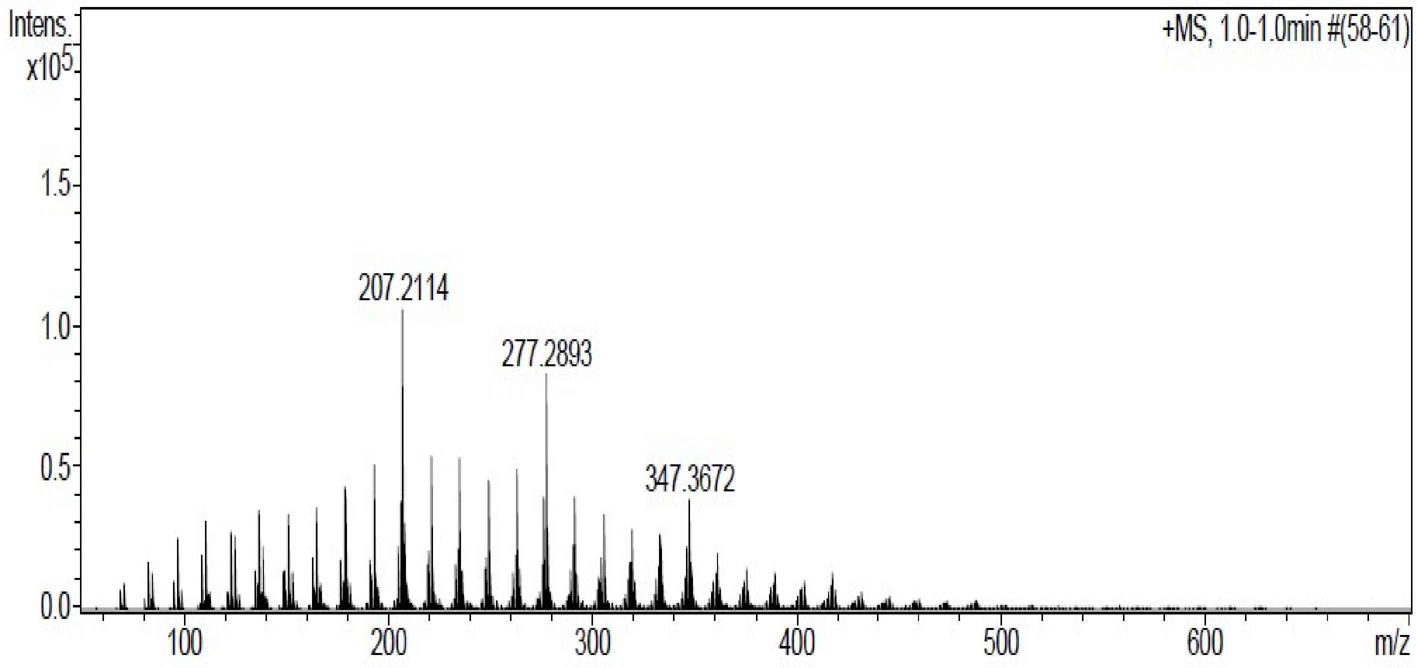

Figure S2: A typical APCI spectrum of the oligomer synthesised using pre-catalyst **1**

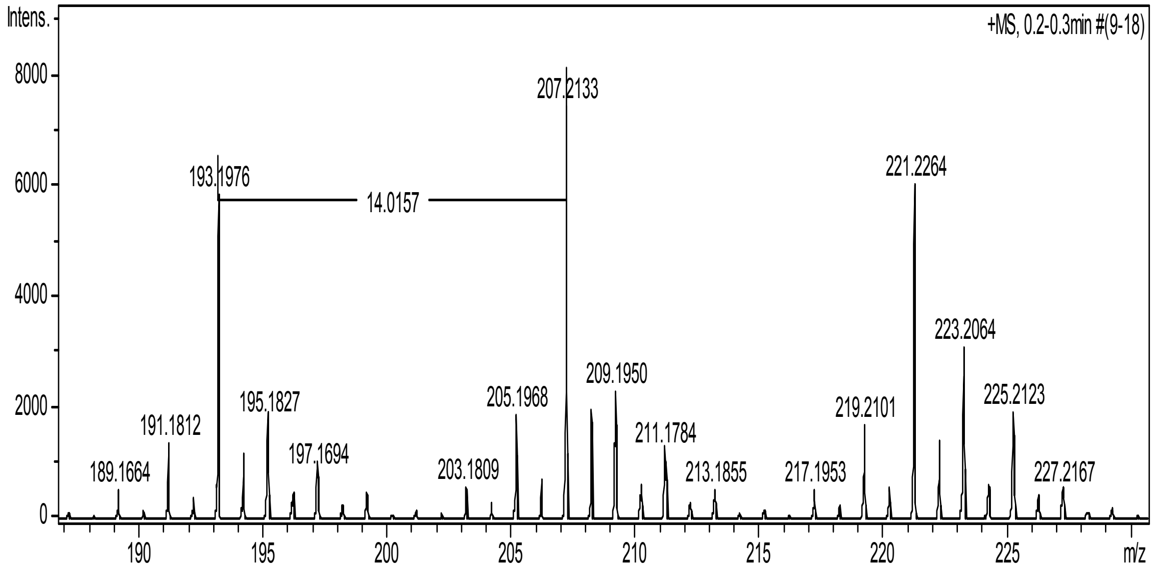

Figure S3: A typical APCI spectrum of the oligomer showing isotopic distributions

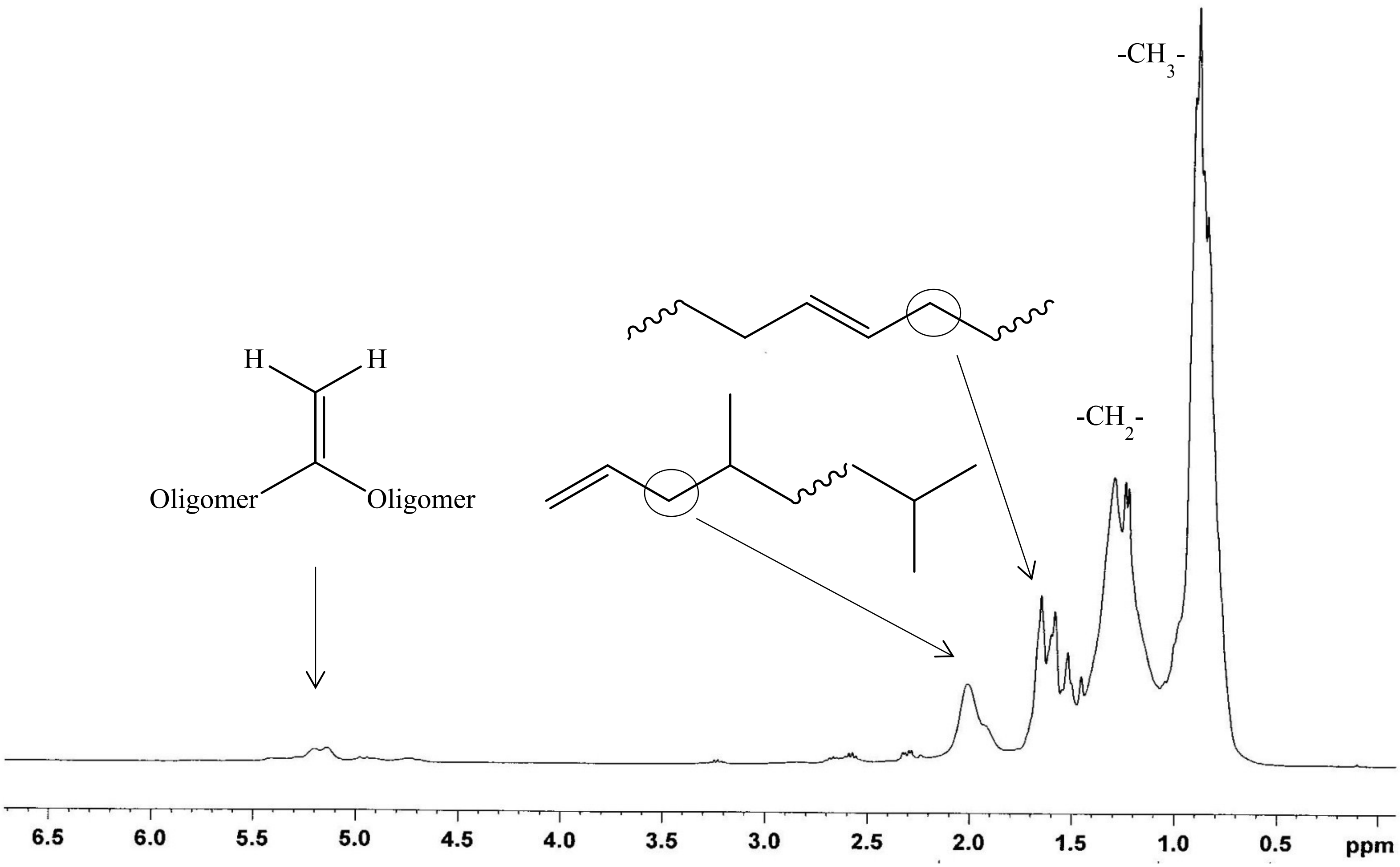

Figure S4: ^{1}H NMR spectrum of ethylene oligomer produced using pre-catalyst **3** run in $CDCl_3$ at 25 ^{o}C

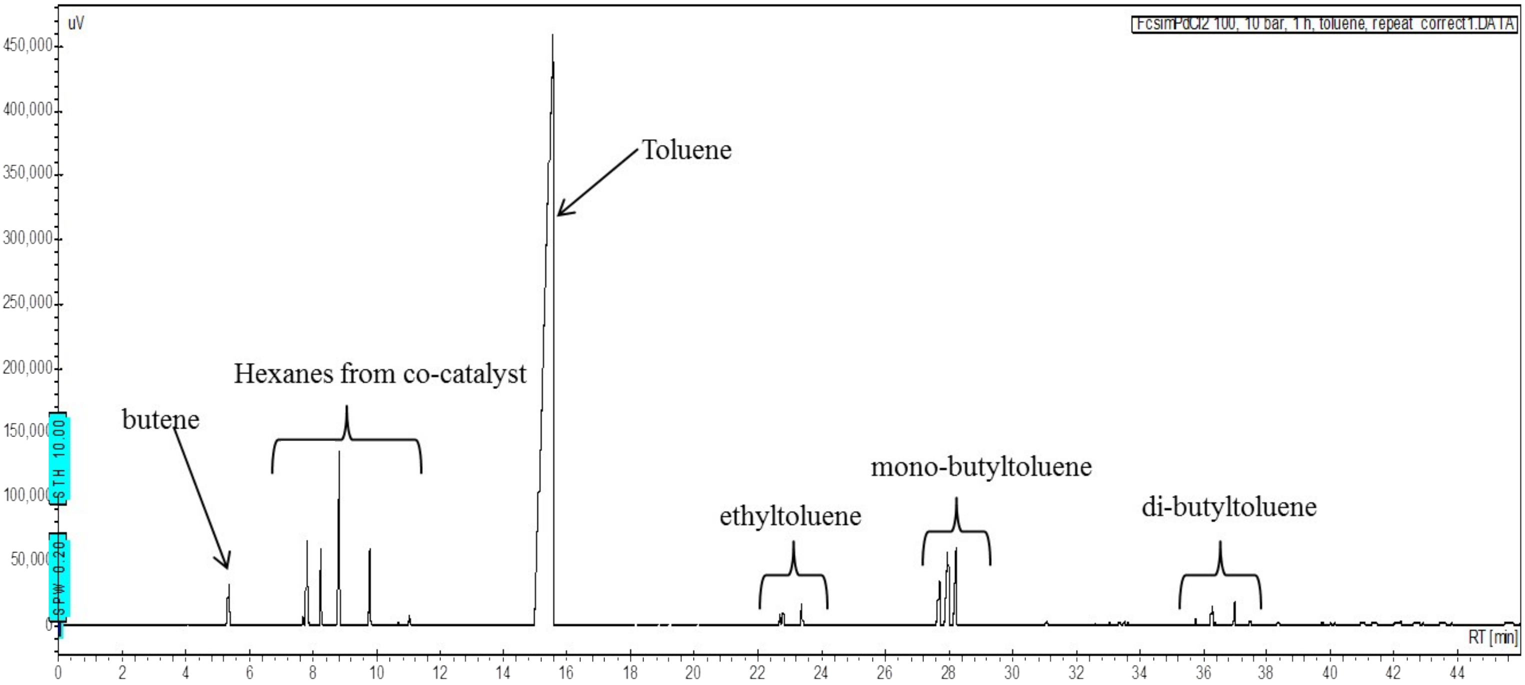

Figure S5: GC showing butene and alkylated toluenes produced using pre-catalyst **3**, $EtAlCl_2$ co-catalyst in toluene solvent, temperature = 25 ^{o}C, Al:Ni =200:1, pressure = 10 bar, time = 1 h.

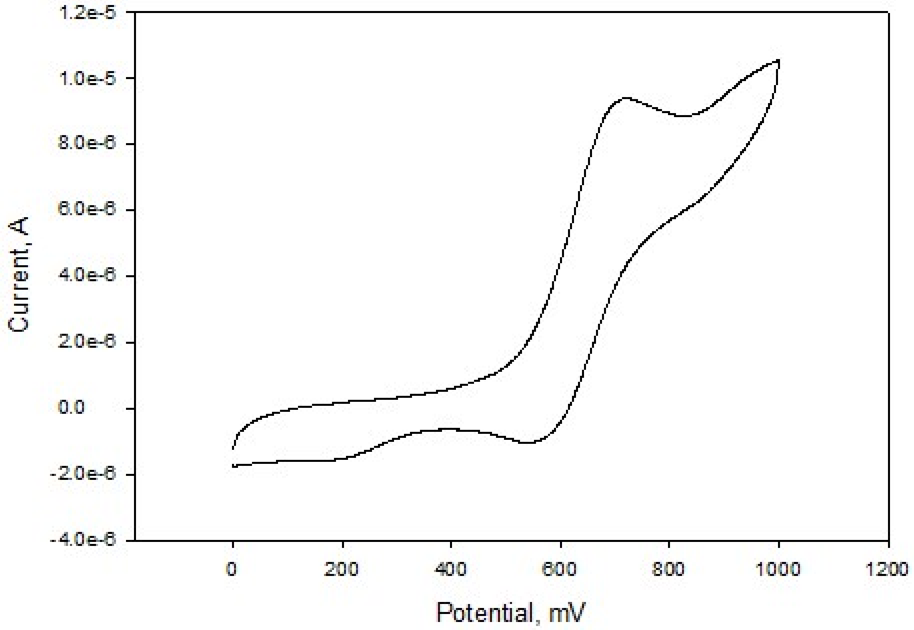

Figure S6: Cyclic voltammogram of compound **L7** in DMF, [^{t}BuN][BF_4] as supporting electrolyte, Ag/AgCl as reference electrode, platinum wire as counter electrode, glassy carbon as working electrode, scan rate of 100 mVs^{-1}, temperature 25 ^{o}C.

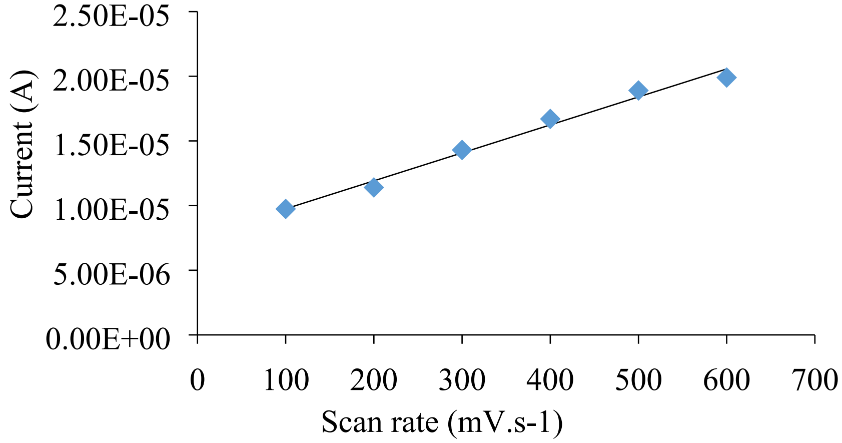

Figure S7: A plot of scan rate *vs*. current for ligand **L2** showing diffusion control mechanism

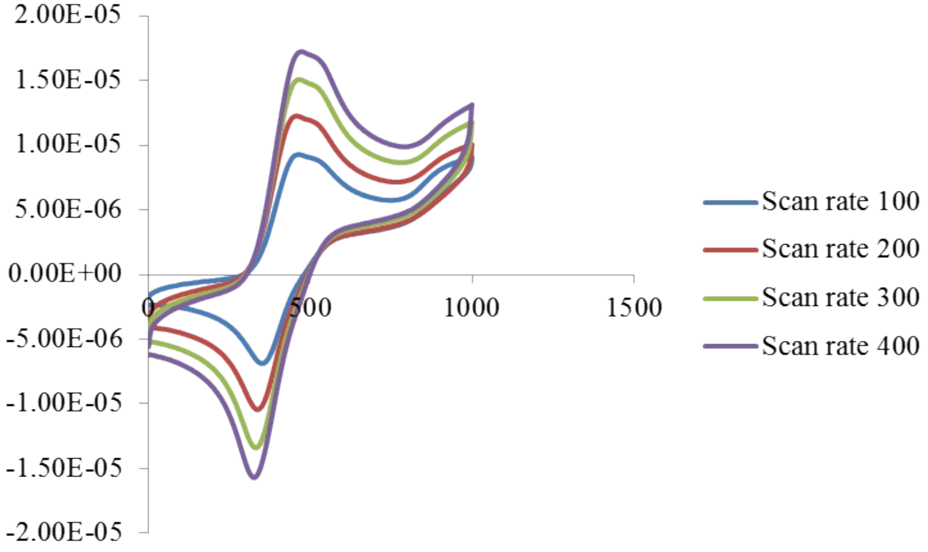

Figure S8: Cyclic voltammogram of compound **L2** with different scan rates showing independent of formal oxidation potential with scan rates (mV.s^{-1}).

Table S1: Half wave potentials and catalytic activity of pre-catalysts **1-8**

Entry	Compound	$E_{1/2}$ (mV)	Activity (kg.molNi^{-1}.h^{-1})
1	**1**	425.0	262
2	**2**	393.0	196
3	**3**	436.0	210
4	**4**	433.0	130
5	**5**	425.0	1 776*
6	**6**	393.0	1 023*
7	**7**	436.0	671*
8	**8**	433.0	578*
9	**9**	417.0	823*
10	**10**	424.0	710*

Activity obtained under the condition; ethylene pressure = 10 bar, Al:Pd = 200, time = 1 h, solvent = hexane. Activity obtained under the condition; ethylene pressure = 10 bar, Al:Ni = 400, time = 1 h, solvent = chlorobenzene.* Values obtained from reference 13b.

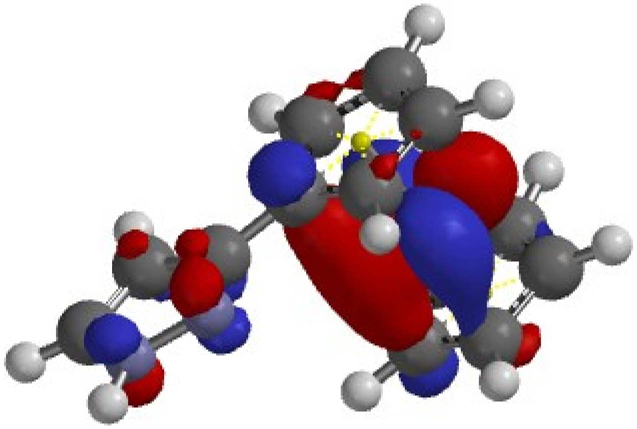

L1 HOMO

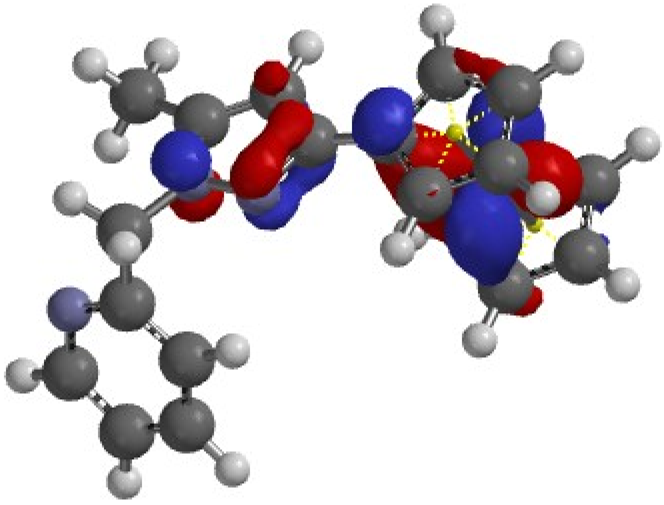

L4 HOMO

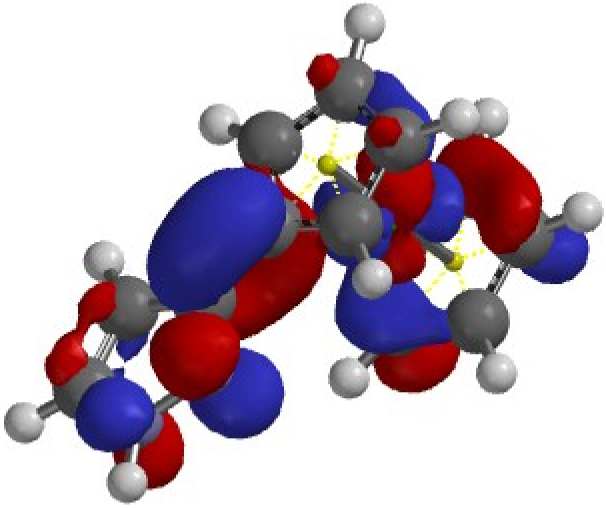

L1 LUMO

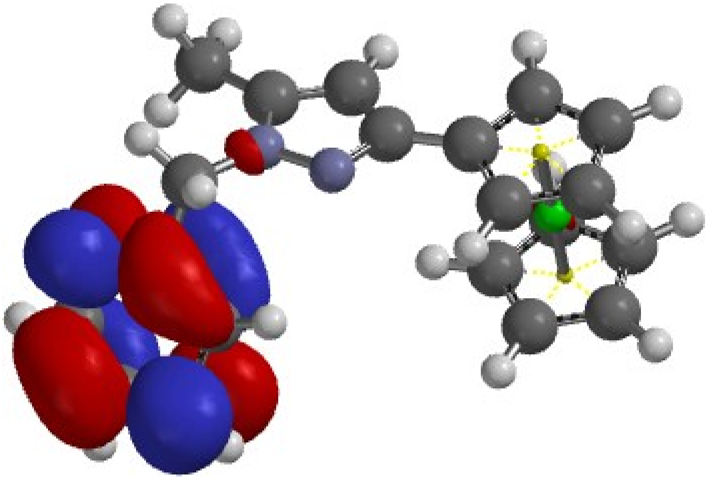

L4 LUMO

Figure S9: Shapes of HOMO and LUMO of ligands **L1** and **L4** using B3LYP level of theory and LACVP* basis set

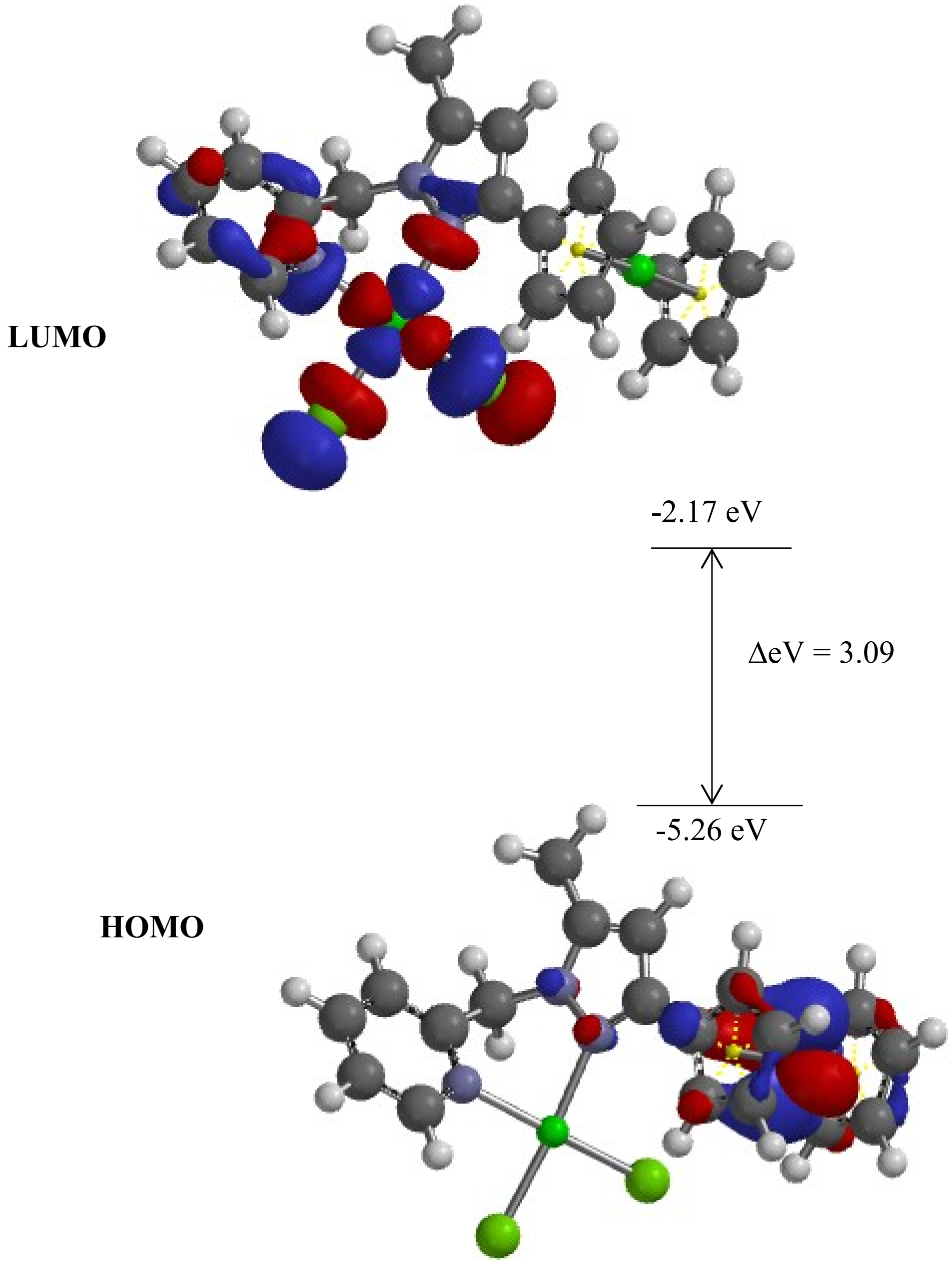

Figure S10: Frontier molecular orbitals of palladium complex **2** as determined by density functional theory using B3LYP level of theory and LACVP* basis set with isovalue of 0.032

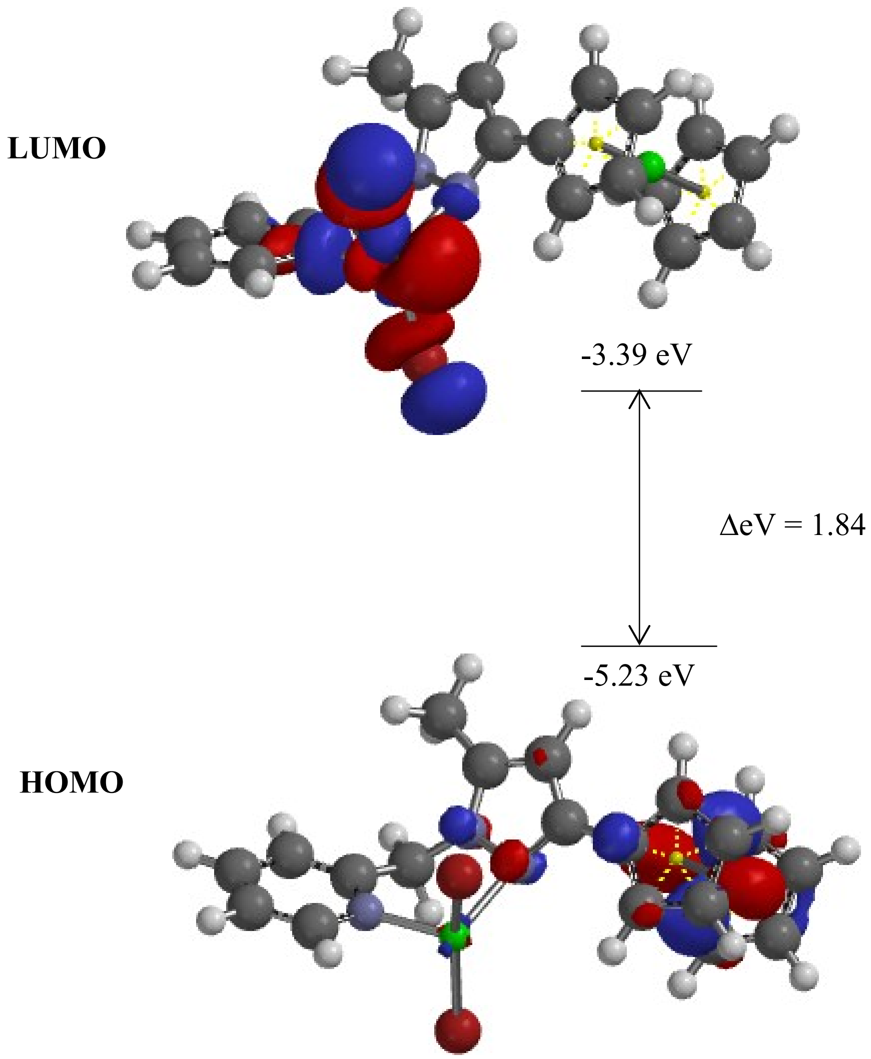

Figure S11: Frontier molecular orbitals of nickel complex **6** as determined by density functional theory using B3LYP level of theory and LACVP* basis set with isovalue of 0.032

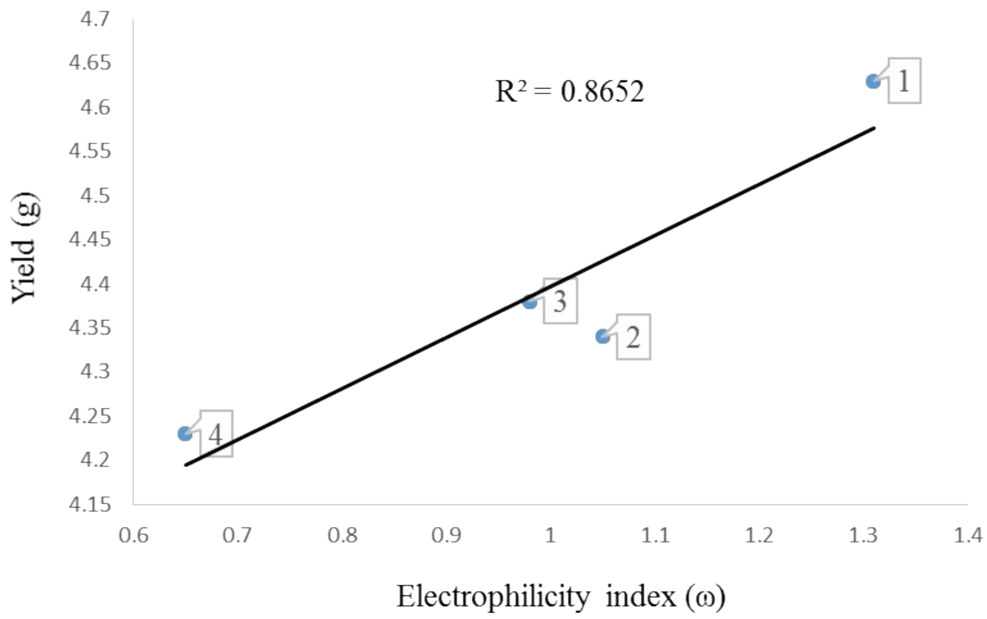

Figure S12: A graph of electrophilicity index against yield of oligomers produced using complexes **1-4**

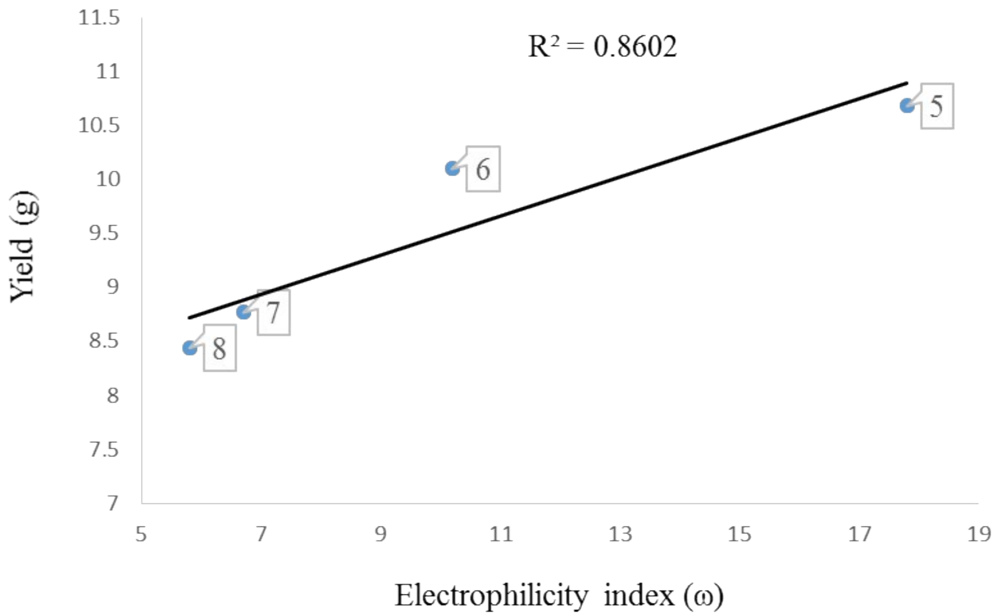

Figure S13: A graph of electrophilicity index against yield of oligomers produced using complexes **5-8**

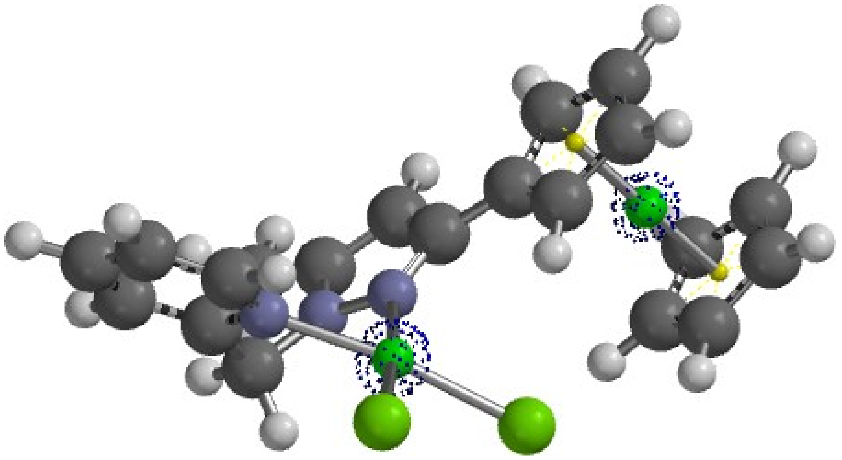

Figure S14: Molecular modelling structure of the electron density of nickel in **1** as determined by density functional theory using B3LYP level of theory and LACVP* basis set with isovalue of 1.0

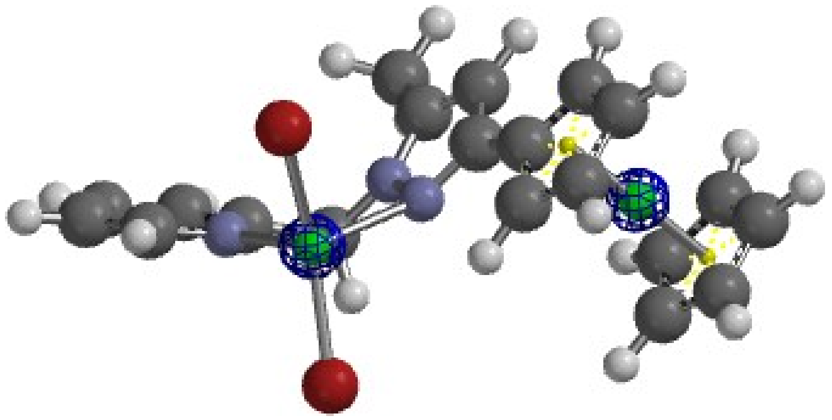

Figure S15: Molecular modelling structure of the electron density of nickel in **6** as determined by density functional theory using B3LYP level of theory and LACVP* basis set with isovalue of 1.0

Table S2: Electron densities of palladium in complexes **1-8**

Complex	Charge density	Yield (g)*
**1**	0.98547	1.31
**2**	0.98409	1.05
**3**	0.98367	0.98
**4**	0.98301	0.65
**5**	1.16578	17.80
**6**	1.16443	10.20
**7**	1.16321	6.70
**8**	1.16232	5.80

Yield obtained under the condition; ethylene pressure = 10 bar, Al:Pd = 200, time = 1 h, solvent = chlorobenzene. Yield obtained under the condition; ethylene pressure = 10 bar, Al:Ni = 400, time = 1 h, solvent = chlorobenzene.* Values obtained from reference 13b.
